# Accurate Spectral Estimation Technique Based on Decimated Linear Predictor for Leak Detection in Waterworks

**DOI:** 10.3390/s21062185

**Published:** 2021-03-20

**Authors:** Aimé Lay-Ekuakille, Vito Telesca, Paolo Visconti, Nicola Ivan Giannoccaro

**Affiliations:** 1 Department of Innovation Engineering, University of Salento, 73100 Lecce, Italy; paolo.visconti@unisalento.it (P.V.); ivan.giannoccaro@unisalento.it (N.I.G.); 2 School of Engineering, University of Basilicata, 85100 Potenza, Italy; vito.telesca@unibas.it

**Keywords:** leak detection in waterworks, pipeline hydrodynamics monitoring, spectral estimation in sensed signals, agricultural water distribution control, pressure sensors

## Abstract

Rural pipelines dedicated to water distribution, that is, waterworks, are essential for agriculture, notably plantations and greenhouse cultivation. Water is a primary resource for agriculture, and its optimized management is a key aspect. Saving water dispersion is not only an economic problem but also an environmental one. Spectral estimation of leakage is based on processing signals captured from sensors and/or transducers generally mounted on pipelines. There are different techniques capable of processing signals and displaying the actual position of leaks. Not all algorithms are suitable for all signals. That means, for pipelines located underground, for example, external vibrations affect the spectral response quality; then, depending on external vibrations/noises and flow velocity within pipeline, one should choose a suitable algorithm that fits better with the expected results in terms of leak position on the pipeline and expected time for localizing the leak. This paper presents findings related to the application of a decimated linear prediction (DLP) algorithm for agriculture and rural environments. In a certain manner, the application also detects the hydrodynamics of the water transportation. A general statement on the issue, DLP illustration, a real application and results are also included.

## 1. Introduction

Water distribution in rural waterworks is intended, here, with the objective for agricultural utilization; hence, leaks in pipelines should be considered to avoid loss along the networks. One should think about the context in the quality of this water and the context of where the pipelines are located. For the first issue, water quality, based on the tertiary process, is the main objective of many countries and cities. The availability of treated wastewater is connected to population distribution in a specified area or country. It is acceptable in terms of costs to distribute wastewater in community areas with greater than 100,000.00 inhabitants to minimize costs. The increase in tertiary treated wastewater [1] favors its subsequent use as effluent in agriculture, city washing needs and in other activities such as golf. These applications do not need unrestricted irrigation after tertiary treatment. Irrigation methods generally used in intensive farming are the sprinkler irrigation method [2] and the furrow one. The use of sprinklers is critical to avoid contamination of close soil in the event of low quality of water. Moreover, spraying favors potential contamination of the aerial parts of plants with any eventual pathogenic elements present in the treated wastewater, with possible increased risk to the humans and animals using them.

The second issue, related to the context of where waterworks are located, is also crucial; arid and desert areas, even rural, need to be supplied by water for agricultural utilization. Hence, pipelines should be installed to convey water from production areas to distribution ones. As a general consideration, given the aforementioned premises, whatever the kind of water to be distributed, a strong and robust monitoring system for detecting leakages is mandatory.

Losses in waterworks can reach enormous values due to the quality of materials employed for their construction; lack of maintenance also has a great impact on leakage. There is no need to explain the importance of water. Therefore, pipelines and waterworks should be monitored [3] to prevent leakage. There are different techniques used for this purpose. For waterworks dedicated to drinkable usages, since the diameter of pipelines can be significant, notably from 0.05 up to 2.00 m, the following techniques can be used: (i) non-invasive methods based on the use of techniques such as terrain analysis for detecting chemical substances; (ii) acoustic emissions [4]; (iii) transient analysis that requires a huge quantity of real-time data, i.e., the elaborate procedure presented in [5], where a wavelet analysis is used for analyzing the transient pressure signal of a water transmission/distribution system to locate the main singularities and the pressure wave speed and a Lagrangian model with a Genetic Algorithm is carried out for detecting the presence and location of some leaks; or the Wireless Water Sentinel project in Singapore (WaterWise@SG) [6], an innovative setup for continuous remote monitoring of a water distribution system composed of sensor nodes deployed across the water distribution system that acquire and transmit data via the Internet to the archive server and that can detect transients in pressure traces for leak detection and location by using wavelet analysis; (iv) electromagnetic waves and micro-robots moving inside the pipeline.

For tertiary treated wastewaters, to be distributed, the main pipeline from the tertiary plant generally has a diameter of around 0.20–0.30 m, and the pipeline to be used in farming exhibits a very small diameter, around 0.01 m, for instance. In this case, the technique for monitoring leaks should, hopefully, be based on sensors mounted on pipeline. In this case, pressure sensors can be mounted on the pipeline, pressure fluctuations can be detected and recovered signals can be sent to a central system for processing. That is a more viable solution with less costs. However, suitable techniques should be used for spectral estimation. These techniques, namely fast Fourier transform (FFT) [7], short-term Fourier transform (STFT) [8], decimated signal diagonalization (DSD) [9], filter diagonalization (FDM) [10] and decimated Padé approximant (DPA) [11], act on a finite duration discrete time signal that always exists, and providing only that the values of the signal are finite. For instance, FFT is simply a weighted sum of a finite number of terms and there are no limits. In all of the other cases, the existence of the Fourier transform is not as obvious.

**Figure 1:**
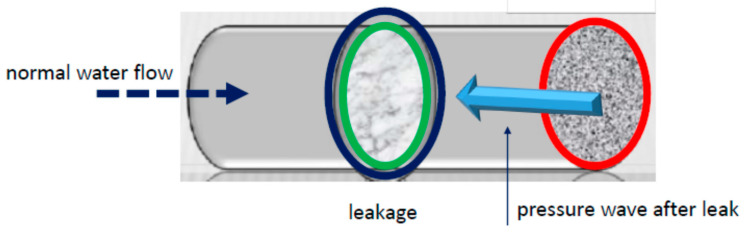
Leakage mechanism after a pressure wave within a pipe.

Leak detection by means of spectral analysis is one of the most important techniques which does not require important instrumentation and equipment. It requires one or more sensors to be mounted on the pipe, allowing to acquire an electric signal that corresponds to the amount of vibrations due to normal liquid flow and sudden peaks connected to leakage. The mechanism of leak detection is explained in Figure 1. The regime of normal flow does not affect the operating mode of the water transportation. In the event of leakage, there is a reverse flow that opposes the normal flow and determines a leak, as reported in the figure. The established transient produces a series of vibrations that are converted to a spectrum. This transient is like a decaying process as it is noticed in nuclear magnetic resonance (NMR) and ionizing radiation. This similitude justifies using algorithms fitted for this process to better recover the signal considering that the pipe is similar to a vein or artery that undergoes thrombosis or a stroke that may affect the regular regime of blood flow. In fact, this is a recent field of research denoted as health hydrology, similar to formal hydrology.

As reported in this paper, FDM, DSD, DPA and decimated linear prediction (DLP) can be of help if we apply the same process. A problem arises in the case of small pipes with different sections. The smaller the section, the more difficult it is to recover the right position of the leakage because the inner height of the pipe does not allow many successive reflections of the waves within the pipe. Hence, a low height works as an attenuator. Conventional techniques such as FFT and STFT exhibit ascertained evidence of low accuracy, low precision and major uncertainty more than the aforementioned techniques.

**Figure 2:**
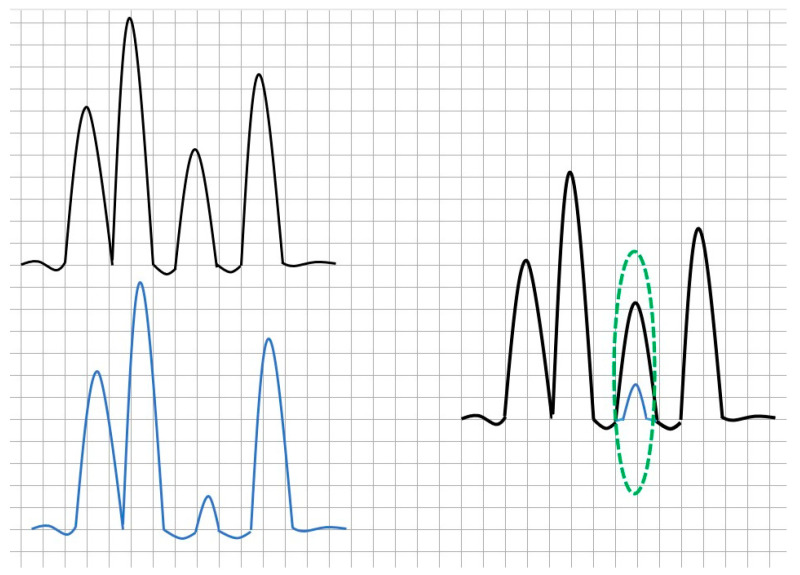
Spectral detection of leak: top left, the theoretical spectrum, and bottom left, the actual detected spectrum; the right feature is the comparison of previous spectra leading to leak detection.

It is related to the lower accuracy in the recovery of the peak, which is less pronounced due to the lower amplification of the wave; this situation can be interpreted as a higher percentage of noise in the spectrum. Figure 2 illustrates the summation and then the comparison between two different series of waves. The first (top left) is the theoretical spectrum, simulated as it is described in the testing section, to be overlapped with the second one (bottom left) so that the result brings overlapped spectra, depicted in the right. One can see that there is no coincidence or total overlapping in a specific window. This lack of overlapping is exactly the leak. The distance between the lower peak and the higher peak in the circled window is a strong indicator of the noise included in the processing. For the conventional techniques such as FFT, TFT, wavelets, etc., this distance is higher than in the newer aforementioned techniques. In practice, this means resolution and immunity to noise.

The DLP technique is suitable for rural and agricultural pipelines, i.e., for pipelines exhibiting a small diameter or section and low flow rate, because DLP works very well in these conditions. The lowest diameters of these pipelines, especially for final distribution, could range from around 4 (0.157 inches) up 25.4 mm (1 inch). They are equipped with sprinklers and the material is generally rubber, polymer and sometimes metal. Certainly, as it is mentioned in the references, advanced transforms can be used in all pipelines transporting water. Water, especially in Europe, is used for urban, industrial, energy and agriculture uses. The latter plays an important role in terms of the amount and percentage. The waterworks for agriculture cover the lion’s share [12].

In this paper, this new and more refined technique, the decimated linear predictor (DLP), is introduced and tested, demonstrating its efficiency and usefulness on an experimental benchmark.

## 2. Decimated Linear Predictor

Regardless of the spectral approach, as numbered above, it is a question of processing signals from a sensor mounted on a pipe to monitor pressure and leakage. Therefore, spectral representation is useful when it is necessary to obtain information from a spectral function $f\left(\hat{\Omega }\right)$ of an operator $\hat{\Omega }$ for which eigenvalues and eigenvectors are known:

(1) $$f\left(\hat{\Omega }\right)=\sum_{k}f\left(\omega_{k}\right)|\omega_{k})(\omega_{k}|$$

The function $f\left(\hat{\Omega }\right)$ is also an operator, with eigenvalues $f\left(\omega_{k}\right)$ and eigenvectors $\left|\omega_{k}\right)$. To show how the algorithm works, it is necessary to modify the specific transform (FFT, STFT, DSD, FDM, DPA) to be adapted as an algorithm by defining a complex one-dimensional signal in time domain $c_{n}=C\left(t_{n}\right)$, defined in a set of equidistant time intervals $t_{n}=n\tau ,n=0,1,\ldots ,N-1$ as a sum of damped sinusoids [13],

(2) $$c_{n}=\sum_{k=1}^{K}d_{k}e^{-in\tau \omega_{k}}=\sum_{k=1}^{K}d_{k}e^{-2\pi in\tau f_{k}}\cdot e^{-n\tau \gamma_{k}}$$

with a total of 2*K* unknowns; that is, *K* complex amplitudes $d_{k}$ and *K* complex frequencies $\omega_{k}=2\pi f_{k}-i\gamma_{k}$ that also include damping. Although Equation (2) is nonlinear, its solution can be obtained from linear algebra. In general, as for FFT, the proposed transform associates an autocorrelation function, with an appropriate dynamic time system described by a complex symmetric operator $\hat{\Omega }$ with complex eigenvalues $\left\{\omega_{k}\right\}$, to signal $c_{n}$ to be transformed in the form of Equation (1):

(3) $$c_{n}=\left(\Phi_{0}|e^{-in\tau \Omega }\Phi_{0}\right)$$

In this manner, the problem can be simplified as a diagonalization of the operator $\hat{\Omega }$ [14] or, similarly, an evolution operator:

(4) $$\hat{U}=\exp\left(-i\tau \Omega \right)$$

Decimated linear prediction falls within a general concept of linear predictors and prediction that is a wide area of research to be used, in particular, in control systems. The interest here is in predicting the behavior of damped exponential signals (see Equation (1)) captured by sensors due to water flow and the effects of leaks within a pipe. A linear predictor should increase sensitivity [15], for noisy signals, and exhibit increased spectral resolution. That is achieved by a connection between the data and the desired spectral parameters; this yields a linearization of the analytical formulae that describe all detected points included in the treated signal.

A decimation [16] is also important since it impacts the quality of the signal by reducing the sample rate within a certain value—that is, reducing high-frequency components. The problem of solving the below equation,

(5) $$C_{n}=\sum_{k=1}^{K}d_{k}z_{k}^{n}\;\;\;\;\;\;\;\;\;\;\;\;\;\;\;\;n=0,1,\ldots ,2K-1;$$

where the amplitudes *d_k_*, connected to the peaks to be determined, are computed by the linear set of equations expressed in this section, and *z_k_*are zeros of the polynomial expression for allowing to find the solution corresponding to the peak, was tackled in the eighteenth century by Baron de Prony [17], who transformed the system of the nonlinear Equation (5) in a problem of linear algebra. This approach is named linear predictor (LP).

The algorithm proposed in [17], decimated linear prediction (DLP), slightly modifies the LP methodology by replacing the native signal *C^sc^(s) (*the superscript sc indicates the sampled signal) with its homologous *C_n_*≡*C^s^*^c^_bl_(*nτ)* obtained by a band-limited decimation. It is possible to transform Equation (1) in (6) using matrices and vectors for the signal points *c_n+*1*_*to *c_n+K_*.

(6) $$\left(\begin{array}{c} c_{n+1}\\ \vdots \\ c_{n+K} \end{array}\right)=\left(\begin{array}{ccc} z_{1}^{n+1} & \ldots & z_{K}^{n+1}\\ \vdots & \; & \vdots \\ z_{1}^{n+K} & \ldots & z_{K}^{n+K} \end{array}\right)\left(\begin{array}{c} d_{1}\\ \vdots \\ d_{K} \end{array}\right)$$

Equation (6) may be solved as (7) for predicting the signal point *c_n,_*

(7) $$c_{n}=\left(\begin{array}{ccc} z_{1}^{n} & \ldots & z_{K}^{n} \end{array}\right){\left(\begin{array}{ccc} z_{1}^{n+1} & \ldots & z_{k}^{n+1}\\ \vdots & \; & \vdots \\ z_{1}^{n+K} & \ldots & z_{K}^{n+K} \end{array}\right)}^{-1}\left(\begin{array}{c} c_{n+1}\\ \vdots \\ c_{n+K} \end{array}\right)=\sum_{k=1}^{K}a_{k}c_{n+k}$$

In this way, by using Equation (7), every signal point *c_n_*may be estimated by linearly combining the *K* following points with fixed coefficients *a_k_*, *k = *1*, …, K*.

The LP algorithm starts calculating the fixed coefficients *a_k_*_,_ solving the linear set of Equation (8) obtained by (7) with *n* = 0, …, *K* − 1.

(8) $$\left(\begin{array}{ccc} c_{1} & \ldots & c_{K}\\ \vdots & \; & \vdots \\ c_{K} & \ldots & c_{2K-1} \end{array}\right)\left(\begin{array}{c} a_{1}\\ \vdots \\ a_{K} \end{array}\right)=\left(\begin{array}{c} c_{0}\\ \vdots \\ c_{K-1} \end{array}\right)$$

The second step of the LP algorithm determines the parameters *z_k_* in Equation (5) by substituting Equation (7) in Equation (5) as shown in Equation (9), then rewritten in Equation (10).

(9) $$c_{n}=\sum_{k=1}^{K}a_{k}c_{n+k}=\sum_{l=1}^{K}\sum_{k=1}^{K}a_{k}d_{l}z_{l}^{n+k}$$

(10) $$\sum_{k=1}^{K}\left[\sum_{l=1}^{K}a_{l}z_{k}^{n+l}-z_{k}^{n}\right]d_{k}=0$$

If *z_k_*is a zero of the polynomial indicated in Equation (11), Equation (10) may be satisfied by a generic set of amplitudes *z_k_*_._

(11) $$\sum_{l=1}^{K}a_{l}z^{l}-1=0$$

The frequencies $\omega_{k}^{'}$ and, therefore, the corresponding parameters *z_k_* = exp(-*iω’_k_τ)* may be calculated by evaluating the polynomial zeros in Equation (12).

(12) $$\omega_{k}^{'}=\frac{1}{\tau }\log\left(z_{k}\right)$$

The solution of Equation (12) is the only nonlinear step of the algorithm and it is obtained by using well-known numerical routines for estimating polynomials’ roots. Finally, the amplitudes *d_k_* are calculated by the linear set of equations expressed in Equation (13).

(13) $$c_{n}=\sum_{k=1}^{K}d_{k}z_{k}^{n}\;\;n=0,\ldots ,K-1$$

## 3. Leak Detection in Waterworks: Case Study

**Figure 3:**
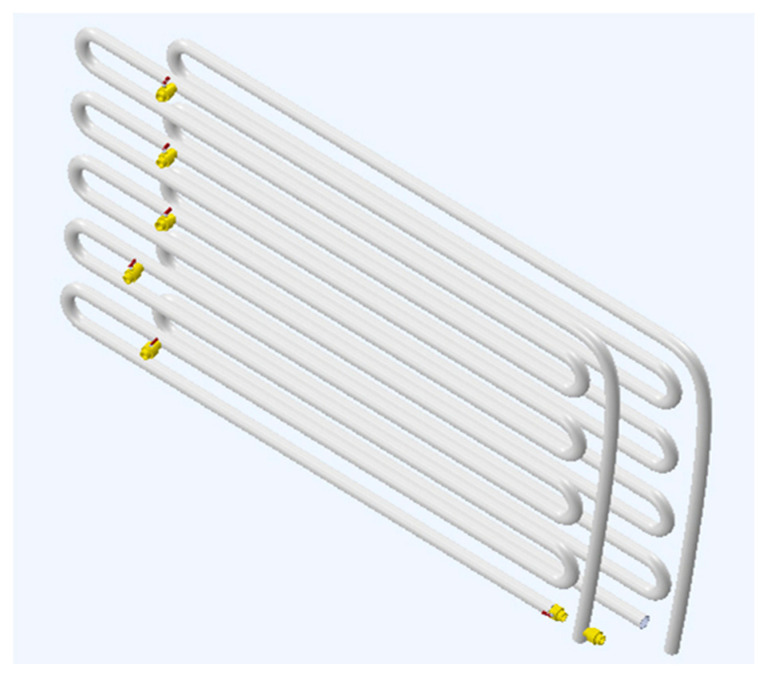
Partial view of zig-zag pipeline, first part of 70 m and back part of 50 m, serving as a hydraulic circuit for leak detection.

Detection of leakage through spectral analysis using sensor data processing is applied here to an experimental and zigzag hydraulic circuit hanged on a wall at a laboratory (Measurement and Instrumentation Laboratory, University of Salento, Italy). The plant consists of two juxtaposed circuits (see Figure 3): the first is for 70 m, and the second for 50 m. The hydraulic circuit has 11 water taps for simulating leaks. These water taps are opened and closed manually. The architecture of the experimental plant reflects an extreme case of common waterworks and water distribution systems for drinking water and agricultural uses, respectively. In fact, it is composed of two main parts: an ascending part for around 70 m, and a descending one for 50 m. Both parts are zigzagged as a serpentine with a section of 1 inch. The pipes are fixed on the wall by means of a bracket with a little stack to allow the pipe to vibrate. As indicated before, this is an extreme configuration. There is a permanent vibration that, even though low, can increase when the pipeline, considered free of water at the beginning, is supplied with pumped water, in case we reach the highest vibrations when free of water until the hydraulic circuit is full of water.

**Figure 4:**
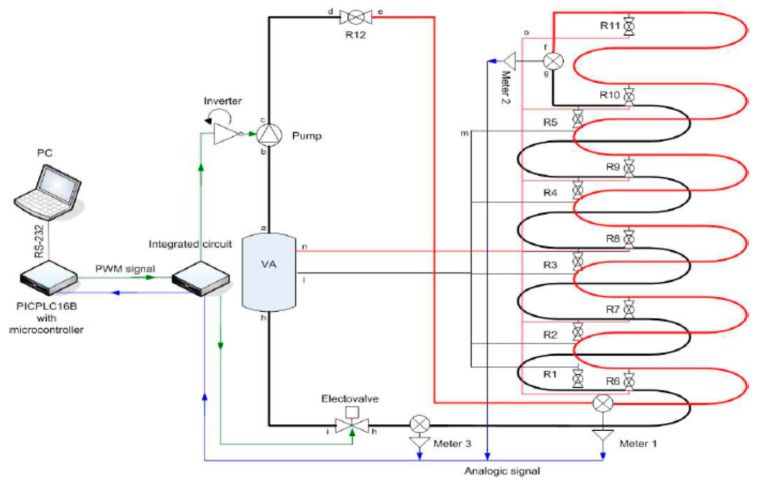
Fully hydraulic circuit/plant with all electronic and mechanical devices.

The plant, as illustrated in Figure 4, contains the following main elements: (i) a 120-m pipeline, manufactured in double stratum-based copper, hanged on a wall of the laboratory; (ii) three pressure sensors at a specific distance; (iii) 11 water taps serving as intentional leaks along the pipeline; (iv) a water tank; (v) an acquisition system with proper electronics; (vi) a pump with a dedicated inverter for speed variations; (vii) a computer.

**Figure 5:**
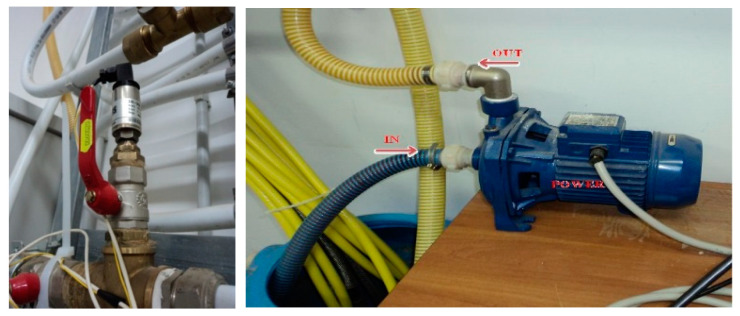
Sensor mounted on pipeline and pump for extracting and launching water in the pipeline. A Gems sensor was used.

The sensors were mounted on the pipe, as shown in Figure 5, with an appropriate valve and joint to allow the exclusion for sensor maintenance and verification. The sensors [18] were connected to acquisition apparatus and their electronics. The sensors capture vibrations from water flow, pushed by the pump (Figure 5, right), to be transduced into an electric signal that corresponds to the water pressure. Further explanations are described in [19]. Since drainers or drippers are often used in many agricultural and rural environments, the collector pipeline’s final section for dispensing was 1 inch or less. The abovementioned configuration exactly reflects the pipeline used for the above environments in buried or surface configuration [20,21,22]. Practically speaking, to run a DLP algorithm, we should use a procedure similar to DPA [23] with a substantial difference given by the computation of amplitudes because DLP employs set of {$\omega_{k}$} computed to solve the system of *K* linear equations focused on the following equations:

(14) cnbld=∑k=1Kdke−jωknτD,Im(ωk)<0

(15) $$c_{n}=\left(\begin{array}{ccc} z_{1}^{n} & \ldots & z_{K}^{n} \end{array}\right)\cdot {\left(\begin{array}{ccc} z_{1}^{n+1} & \ldots & z_{K}^{n+1}\\ \vdots & \ddots & \vdots \\ z_{1}^{n+K} & \ldots & z_{K}^{n+K} \end{array}\right)}^{-1}\;\cdot \left(\begin{array}{c} c_{n+1}\\ \vdots \\ c_{n+K} \end{array}\right)=\sum_{k=1}^{K}a_{k}c_{n+K}.$$

**Figure 6:**
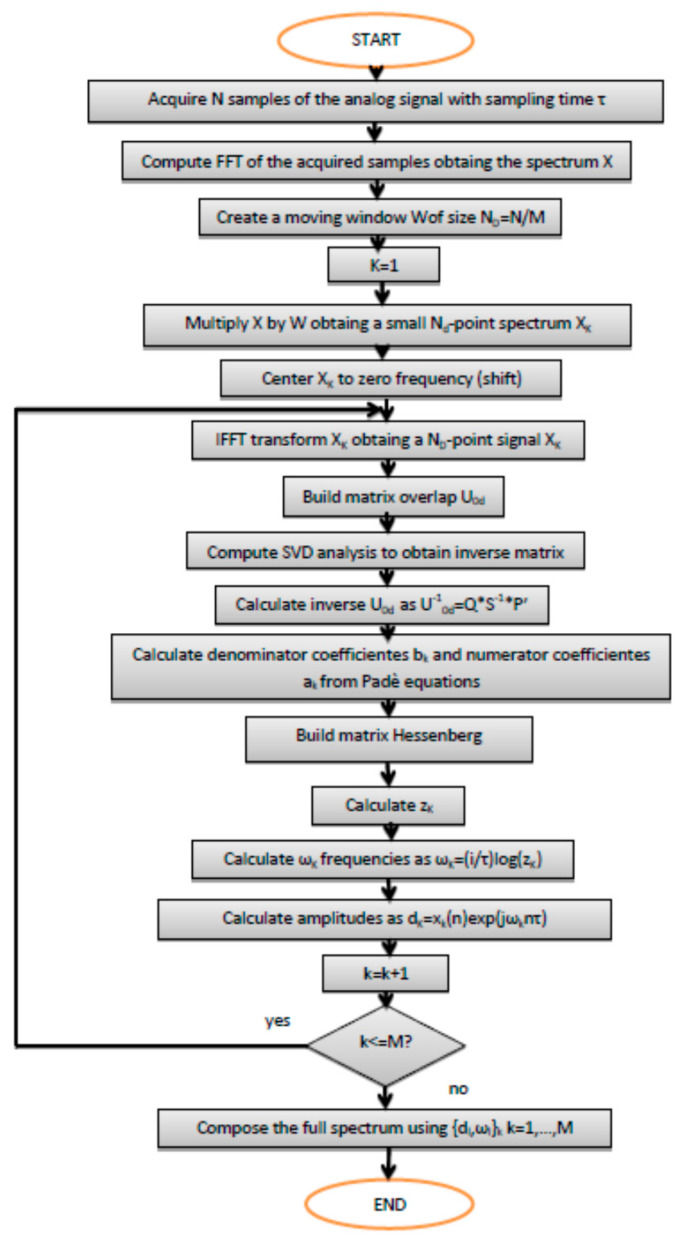
Summary of the decimated linear prediction (DLP) algorithm.

Then, the procedure is here summarized as reported in Figure 6.

**Figure 7:**
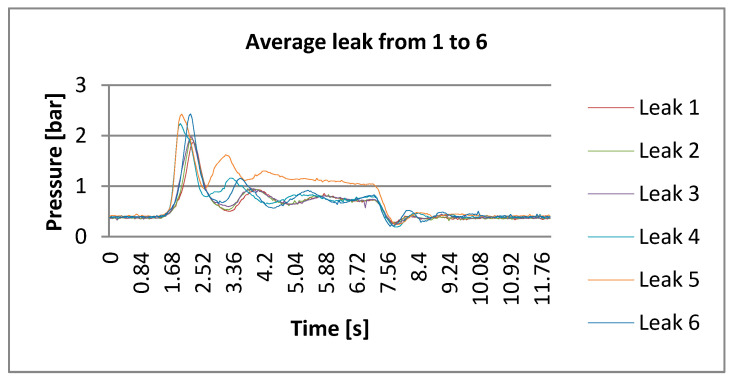
Recorded trends of opening and closing taps for simulating leaks from 1 to 6.

**Figure 8:**
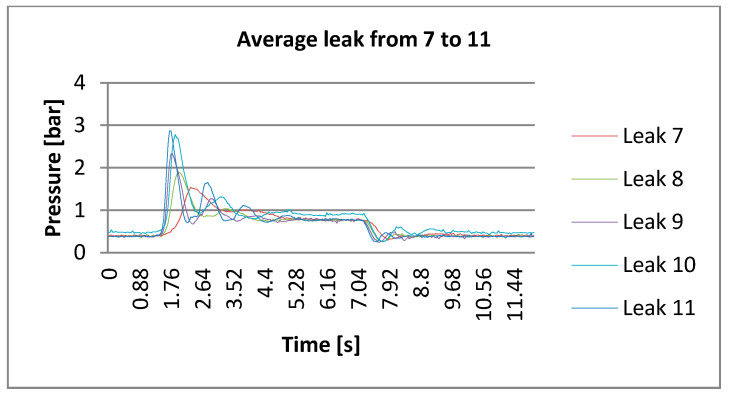
Recorded trends of opening and closing taps for simulating leaks from 7 to 11.

DLP, in practical terms, as per its algorithm, delivers a magnitude response according to the appropriate frequency [24]. Magnitude responses are connected to the opening and closing of valves (water taps). Figure 7 and Figure 8 indicate the trend of the pressure in the time domain during the opening and closing of different taps that simulated leaks. This is the trend of the Poisson distribution and the dynamics of decay process; that is why, a fortiori, the advanced transforms are more suitable, namely DLP. These figures also report the peak during opening and the descending pressures after water tap closing.

**Figure 9:**
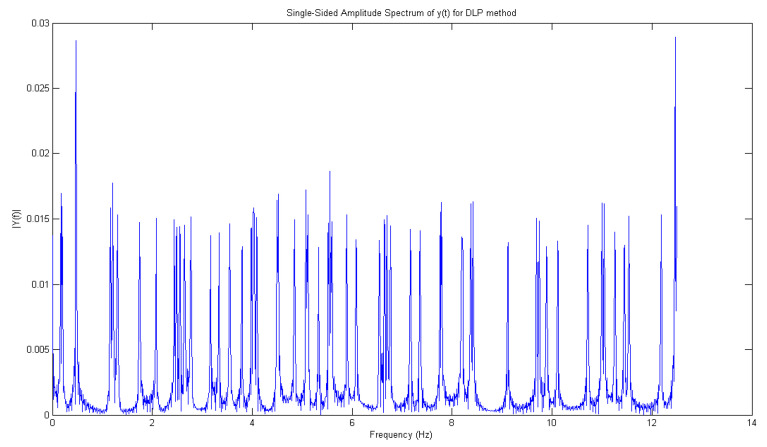
Frequency representation of all peaks (1–6) retrieved from the time domain using the DLP algorithm.

**Figure 10:**
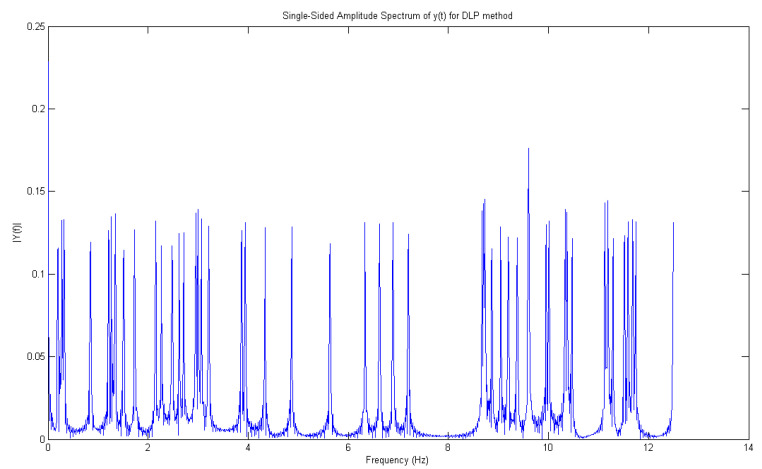
Frequency representation of all peaks (7–11) retrieved from the time domain using the DLP algorithm.

Between 1.4 and 1.5 Hz, according to Figure 9 which represents the results of DLP processing of all signals in the time domain of leaks from 1 to 6, there is an interesting peak, the highest, that enables the best detection of the leak corresponding to water tap opening. The same reasoning is also done for Figure 10.

**Figure 11:**
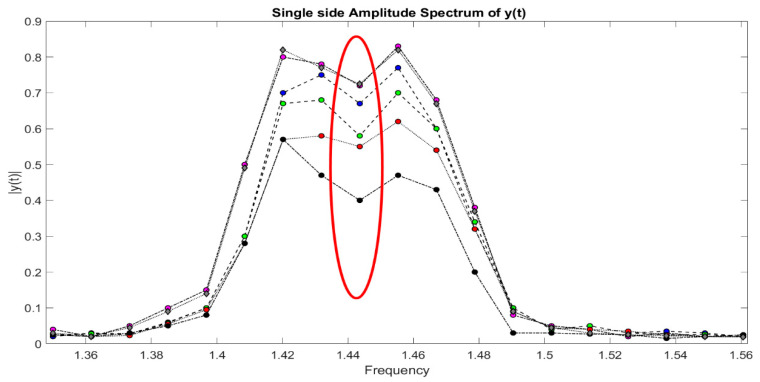
Detected leaks from 1 to 6: magnitude vs. frequency.

**Figure 12:**
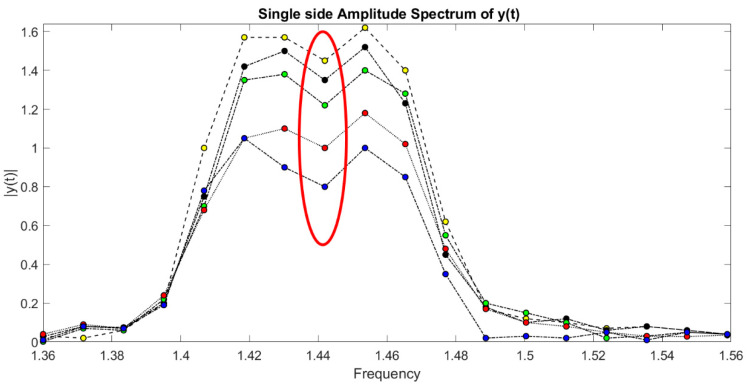
Detected leaks from 7 to 11: magnitude vs. frequency.

From this range, in particular, 1.452 Hz is the right frequency allowing to detect, with major reliability, the leak. Then, as Figure 11 displays, we are able to reveal the right peaks (only these ones) corresponding to the opening of water taps. Since there are two joined zigzag pipelines (one ascending with leaks from 1 to 6 for 70 m), analogously, in Figure 10, the spectrum, through DLP, of the time domain representation of leaks from the 7th up to 11th water tap is reported. Hence, with the detection of leaks, for a descending branch, from the end of the first one, for 50 m to work as one pipeline, DLP exhibits the same range of magnitudes in both cases. The leaks are reported in Figure 12.

This is an important aspect for certain pipelines, explained in the Conclusions section in terms of comparison with other spectral techniques using advanced transforms.

## 4. Results and Discussion

The results shown in Figure 6 and Figure 7 are related to the entire pipeline composed of two joined sections of 70 and 50 m, respectively. They are cognate irrespectively of the magnitudes retrieved at the aforementioned frequency, namely 1.452 Hz. Moreover, to calculate the position of the leak with respect to the close pressure sensor, it is viable to find it using an uncertainty approach. Regression, notably linear and/or quadratic, can help accordingly. In linear regression, for instance, using ordinary least squares [25], it is necessary to calculate coefficients *a* and *b* so that the line of the subsequent equation has a minimum distance to given points:

(16) y=ax+b

Here, the following equations are used:

(17) $$a=\frac{N\sum_{i=1}^{N}x_{i}y_{i}-\sum_{i=1}^{N}x_{i}\sum_{i=1}^{N}y_{i}}{\Delta }\;b=\frac{\;\sum_{i=1}^{N}x_{i}^{2}\sum_{i=1}^{N}y_{i}-\sum_{i=1}^{N}x_{i}\sum_{i=1}^{N}x_{i}y_{i}}{\Delta }$$

(18) $$\Delta =N\sum_{i=1}^{N}x_{i}^{2}-{\left(\sum_{i=1}^{N}x_{i}\right)}^{2}$$

where (*x_i_,y_i_*) are co-ordinates of the poin,; *σ_y_* is the uncertainty standard deviation on values of *y* and *σ_x_* is the uncertainty of *x*, pursuant to the following equations:

(19) $$\sigma_{y}=\sqrt{\frac{1}{N}}\sum_{i=1}^{N}{\left(y_{i}-b-ax_{i}\right)}^{2}\;\sigma_{x}=\left|\frac{dx}{dy}\right|\sigma_{y}=\frac{1}{a}\sigma_{y}$$

The uncertainty permits to define the range [−*σ_x,_σ_x_*], centered on *x*_0_ of values of the distance at which the leak is situated; *x*_0_ is the estimated distance through the used (in this case, linear) model of regression. The lower the uncertainty is, the easier it is to detect the leak position. The formula

(20) $$x_{0}=\frac{y_{0}-b}{a}$$

represents the estimation of the distance from the leak with respect to the sensor position and is the characteristic amplitude of pressure signal spectrum.

**Figure 13:**
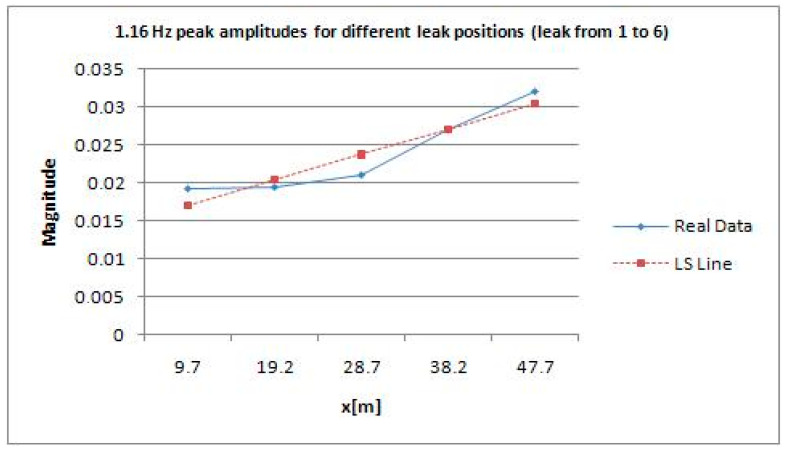
Example of linear regression implementation for retrieving leak position (leak from 1 to 6).

**Figure 14:**
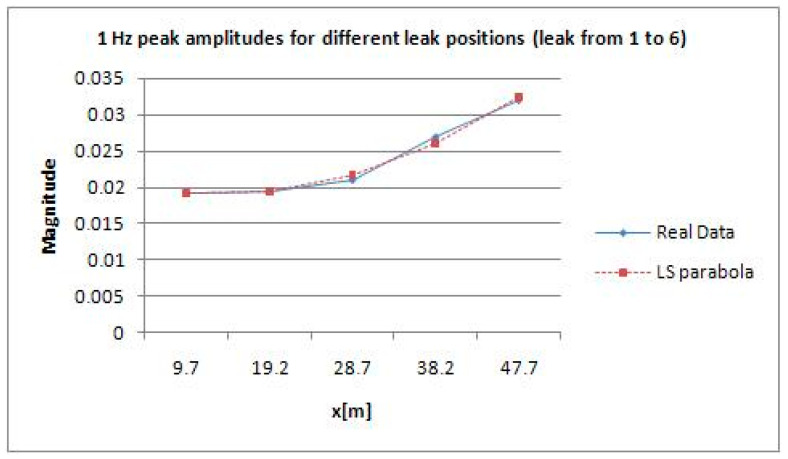
Example of quadratic regression implementation for retrieving leak position (leak from 1 to 6).

Here are two examples of using linear and quadratic regressions for retrieving the position of a leak and its uncertainty. The latter does not take into account the uncertainty of the 2200 A Gems sensors mounted on the pipe. It is neglected because of its low impact on the general calculation since its main features are an output accuracy of 0.15–0.25%, full scale, and a thermal error of 1–1.5%, full scale. Figure 13 and Figure 14 depict the cases of linear and quadratic regressions, respectively; LS stands for least squares.

Table 1 exhibits the summarized results taking into account uncertainty per position. Of course, based on the type of regression, the results change; in particular, for the ascending section, that is, from leak 1 up to 6, the uncertainty is greater than for the descending segment. This is coherent with the architecture of the plant; the pipe material is a double layer-based copper with an external coat of polymer. Therefore, the stiffness of the pipe is much higher than for that used in agriculture for the final distribution. The second effect is that the ascending branch exhibits more friction and possible turbulence than the descending branch.

## 5. Conclusions

The goal of the paper was to demonstrate the usefulness of spectral analysis techniques based on advanced transforms, namely FDM, DSD and DPA, to increase precision and accuracy in retrieving leak positions and to control hydrodynamics in rural pipelines utilized for liquid transportation, notably in agriculture. Each advanced transform possesses its proper specificity, allowing to detect leakage along a pipe. Certainly, for rural pipelines not under roads—hence, with no physical noise impacting on signal acquisition—DLP is suitable given that there are no sudden variations in pressure. That is the case of rural waterworks, pipelines conveying water for parks, green areas to be preserved and agricultural uses. That is normal since DLP is a linear predictor working with linear procedures or a less nonlinear process. In general, with the same pipeline, meaning at the same conditions, DLP lasts, as DPA, with more computational time [26] than FDM and DSD; whether the impact of noise can be neglected or not, they can solve other issues such as wiggles on the spectrum. DLP, as DPA, exhibits a high resolution. DLP is suitable [27] for the case of rural-based water distribution. However, in case of big waterworks or pipelines for water needing huge amounts of data, statistical tests during modeling should be included, for example, multivariate index analysis [28], to check data correlation.

**Figure 15:**
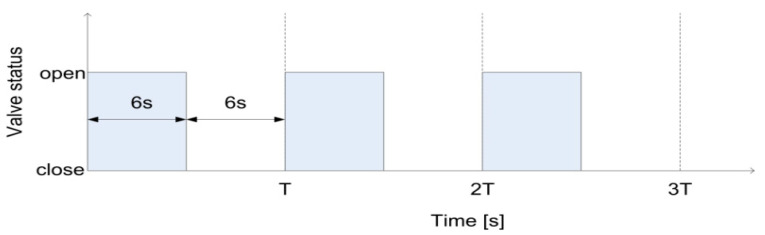
Electrovalve opening and closing to modify the flow conditions.

Any detection algorithm, especially based on spectral analysis, relies on uncertainty in revealing the correct position, accuracy, spectral resolution and the time between the leak occurrence and its detection—that is, the response time. The latter, on equal terms with respect to FDM, DSD and DPA, is also a critical parameter. To understand this reasoning, Figure 15 traces out the time of the opening and closing of the electrovalve after the pump that allows to modify the flow conditions; 12 s is the duration of opening and closing. The pump (an Ebara CMA 0.50M [29]) displays a maximum pressure of 6 bar and a flow rate ranging from 0.333 up to 2.333 L/s. Conversely, the sensor measurement range is 0–10 bar.

**Figure 16:**
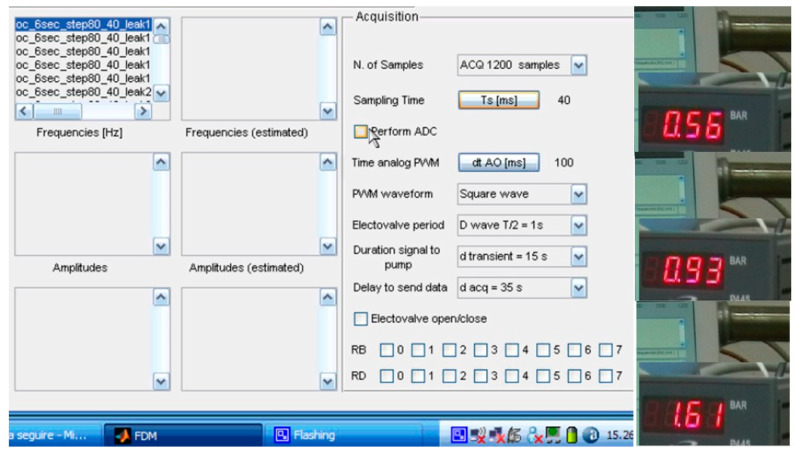
MATLAB-based program shell for performing the acquisitions according to different parameters and advanced transforms.

Having fixed the flow conditions as per Figure 15, the acquisition parameters, as reported in Figure 16, can vary; hence, the pressure and the flow rate influence the response time. Given 0.65 L/s as the flow rate and up to 0.93 bar, DLP exhibits the shortest response time around 3–5 s that lays in the interval of 12 s of opening and closing, while the DPA displays 5–7 s, and the response times of FDM and DSD are major. However, after 0.93 bar and growing near 1.61 bar and further up to 3 bar, and with a flow rate close to the maximum that the pump can allow, particularly in case of recurrent opening and closing, mimicking major turbulence, DLP displays the worst response time, reaching 3–4 times the previous one. The best response time, in this case, is yielded by DSD, regardless of the spectral resolution.

The time response is not a moot point or a captious remark; it is also a key issue, especially in agriculture, when drainers or drippers are used for long distances. In extensive and intensive plantations where the pipelines can reach dozens of km, the response becomes crucial. In general, as we may know, in agricultural applications, a single user can be considered not only as an obvious passive hydraulic load but also with an almost constant flow rate. Thus, DLP is more suitable, as we can reaffirm, than the other advanced transforms.

The proposed approach renders the interaction sensor and the proposed technique as a “smart process for an intelligent sensing”. In this context, the technique proposed can also be used by combining remote sensing data and hydrological modeling to optimize irrigation procedures [30].

## Figures and Tables

**Table 1:** Recovered leak positions and uncertainty.

	Work Frequency (Hz)	Magnitude	Distance (m)	DLP Uncertainty with Linear Regression (m)	DLP Uncertainty with Quadratic Regression (m)
Leak 1	1.16	0.6651	9.7	±4.75	±8.85
Leak 2	1.16	0.6945	19.2	±4.75	±8.85
Leak 3	1.16	0.7144	28.2	±4.75	±8.85
Leak 4	1.16	0.7654	38.2	±4.75	±8.85
Leak 5	1.16	0.8995	47.7	±4.75	±8.85
Leak 6	1.16	0.9065	57.2	±4.75	±8.85
Leak 7	1.16	0.4145	1.85	±2.2	±0.75
Leak 8	1.16	0.5391	9.85	±2.2	±0.75
Leak 9	1.16	0.6235	17.85	±2.2	±0.75
Leak 10	1.16	0.7053	25.85	±2.2	±0.75
Leak 11	1.16	0.7365	33.85	±2.2	±0.75

## Data Availability

Not applicable.

## References

[1] The European Federation of National Water Services, Europe’s Water in Figures: An Overview of the Europe and Drinking Water and Waste Water Sectors, 2017 Edition. https://www.danva.dk/media/3645/eureau_water_in_figures.pdf.

[2] Boyd G., Na D., Li Z., Snowling S., Zhang Q., Zhou P. (2019). Influent Forecasting for Wastewater for Wastewater Treatment Plants in North America. Sustainability.

[3] Marmarokopos K., Doukakis D., Frantziskonis G., Avlonitis M. (2018). Leak Detection in Plastic Water Supply Pipes with a High Signal-to-Noise Ratio Accelerometer. Meas. Control.

[4] Lei H., Huang Z., Liang W., Mao Y., Que P.Y. (2009). Ultrasonic Pig for Submarine Oil Pipeline Corrosion Inspection. Russ. J. Nondestruct. Test..

[5] Meniconi S., Brunone B., Ferrante M., Capponi C., Carrettini C.A., Chiesa C., Segalini D., Lanfranchi E.A. (2015). Anomaly pre-localisation in distribution-transmission mains by pump trip: Preliminary field tests in the Milan pipe system. J. Hydroinform..

[6] Allen M., Preis A., Iobal M., Srirangarajan S., Lim H., Girod L& Whittle A. (2011). Real-time in-network distribution system monitoring to improve operational efficiency. J. AWWA.

[7] Lay-Ekuakille A., Vendramin G., Trotta A. FFT-Based Spectral Response for Smaller Pipeline Leak Detection. Proceedings of the I²MTC–IEEE International Instrumentation and Measurement Technology Conference.

[8] Lay-Ekuakille A., Griffo G., Visconti P., Primiceri P., Velazquez R. (2017). Leaks Detection in Waterworks: Comparison between STFT and FFT with an Overcoming of Limitations. Metrol. Meas. Syst..

[9] Lay-Ekuakille A., Vergallo P. (2014). Decimated Signal Diagonalization Method for Improved Spectral Leak Detection in Pipelines. IEEE Sens. J..

[10] Lay Ekuakille A., Vendramin G., Trotta A. (2009). Spectral Analysis of Leak Detection in a Zigzag Pipeline:A Filter Diagonalisation Method-based algorithm application. Measurement.

[11] Lay-Ekuakille A., Vergallo P., Griffo G. (2013). A Robust Algorithm based on DPA Technique for Processing Sensor Data in Leak detection in Waterworks. IET Sci. Meas. Technol..

[12] Sectoral Use of Water in Regions of Europe. https://www.eea.europa.eu/data-and-maps/figures/sectoral-use-of-water-in-regions-of-europe.

[13] Lay-Ekuakille A., Fabbiano L., Vacca G., Kidiamboko Kitoko J., Bibala Kulapa P., Telesca V. (2018). A Comparison between Decimated Padé Approximant and Decimated Signal Diagonalization Methods for Leak Detection in Pipelines Equipped with Pressure Sensors. Sensors.

[14] Lay-Ekuakille A., Pariset C., Trotta A. (2010). FDM-based Leak Detection of Complex Pipelines: Robust Technique for Eigenvalues Assessment. Meas. Sci. Technol..

[15] Zhu G.-Y., Henson M.A., Megan L. (2011). Dynamic modeling and linear model predictive control of gas pipeline networks. J. Process Control.

[16] Crochiere R.E., Rabiner L. (1981). Interpolation and decimation of digital signals—A tutorial review. Proc. IEEE.

[17] Marple S.L. (1986). Digital Spectral Analysis: With Applications.

[18] Pressure Transducers. https://www.gemssensors.com/pressure/pressure-tranducers.

[19] Lay-Ekuakille A., Vendramin G., Trotta A. (2010). Robust Spectral Leak Detection of Complex Pipelines using Filter Diagonalisation Method. IEEE Sens. J..

[20] Hohn C.H. (1997). Estimating Water Flow from Pipes, Guide A-104. College of Agriculture, Consumer and Environmental Sciences.

[21] Estimating Water Flow From Pipes. https://aces.nmsu.edu/pubs/_a/A104/welcome.html.

[22] Catalog Agriculture 2020. https://www.scarabelli.it/downloads/.

[23] O'Sullivan E.A., Cowan C.F.N. (2008). Modelling room transfer functions using the decimated Pade approximant. Signal Process. IET.

[24] Guan S., Marshall A.G. (1997). Linear Prediction Cholesky Decomposition vs Fourier Transform Spectral Analysis for Ion Cyclotron Resonance Mass Spectrometry. Anal. Chem..

[25] Hawley S., Ali M.S., Berencsi K., Judge A., Prieto-Alhambra D. (2019). Sample size and power considerations for ordinary least squares interrupted time series analysis: A simulation study. Clin. Epidemiol..

[26] Atal B.S. (2006). The History of Linear Prediction. IEEE Signal Process. Mag..

[27] Byeon S., Choi G., Maeng S., Gourbesville P. (2015). Sustainable Water Distribution Strategy with Smart Water Grid. Sustainability.

[28] Giorgio G.A., Ragosta M., Telesca V. (2017). Application of a multivariate statistical index on series of weather measurements at local scale. Measurement.

[29] Ebara Pumps CMA 0.50M. https://www.centrifugal-pump-online.com/acatalog/Ebara-Pumps-CMA_0.50_M-160.html.

[30] Corbari C., Salerno R., Ceppi A., Telesca V., Mancini M. (2019). Smart irrigation forecast using satellite LANDSAT data and meteo-hydrological modeling. Agric. Water Manag..

